# P-1769. Improving Surgical Prophylaxis for Patients with Penicillin Allergy Label

**DOI:** 10.1093/ofid/ofae631.1932

**Published:** 2025-01-29

**Authors:** Sima L Sharara, Kate Dzintars, Eili Klein, Valeria Fabre, Sara E Cosgrove

**Affiliations:** Johns Hopkins Hospital, Baltimore, Maryland; The Johns Hopkins Hospital, Baltimore, Maryland; Johns Hopkins School of Medicine, Baltimore, Maryland; Johns Hopkins University School of Medicine, Baltimore, MD; Johns Hopkins School of Medicine, Baltimore, Maryland

## Abstract

**Background:**

Patients with a penicillin allergy label (PAL) have an increased risk of surgical site infection when receiving a β-lactam alternative for surgical prophylaxis. The updated 2022 Drug Allergy Parameters support the use of cefazolin for patients with a penicillin allergy history including anaphylaxis. We evaluated antibiotic use for surgical prophylaxis in a large academic center.

**Figure:**
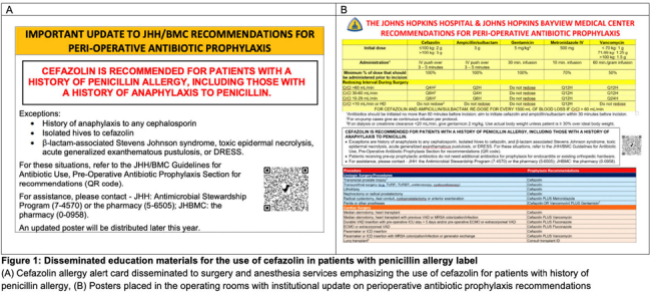

**Methods:**

We performed a quality improvement intervention to decrease inappropriate use of non-β-lactam antibiotic surgical prophylaxis in patients with PAL at a 1091-bed tertiary hospital. Beginning in 10/2018, we provided education at anesthesia grand rounds and surgical safety meetings regarding preferential use of cefazolin in patients without severe PAL. In 12/2023, we updated institutional guidelines to recommend cefazolin use in all patients except those with history of anaphylaxis to cephalosporins, isolated hives to cefazolin, and β-lactam-associated severe cutaneous drug reactions. This was communicated to anesthesia and surgery stakeholders. Peri-operative antibiotic prophylaxis alert cards were hung in the operating rooms (Figure 1) and clindamycin and gentamicin were removed from OR medication-dispensing machines (ORMD).

**Figure:**
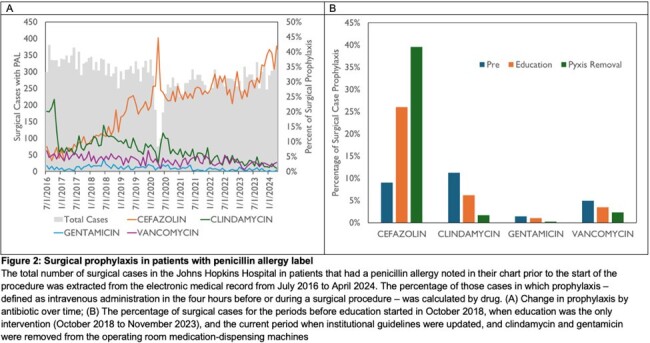

**Results:**

There was a significant decrease in clindamycin use from 11.2% in the pre-education period to 6.2% after education, further reduced to 1.7% since updating institutional guidelines and removing clindamycin and gentamicin from ORMD. Concurrently, cefazolin use increased from 9.0% to 26.0% to 39.5% in the same periods (Figure 2). This was seen across surgical services (Figure 3). Decreases were also seen in gentamicin use. There was no significant change IV vancomycin use as an alternative for patient with PAL. There was no increase in episodes of perioperative anaphylaxis related to these interventions.

**Figure:**
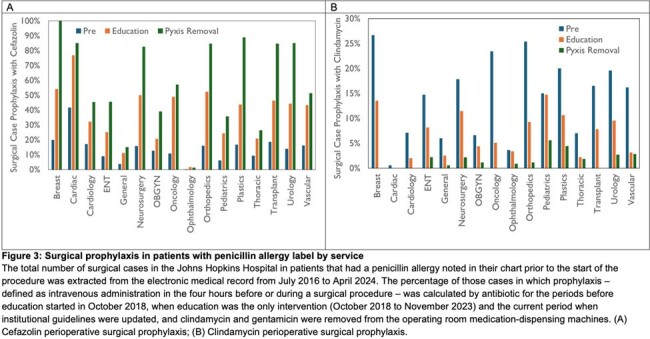

**Conclusion:**

There remains significant opportunity to optimize surgical prophylaxis for patients with PAL. QI efforts such as acquiring stakeholder support and development and dissemination of teaching materials were effective in reducing suboptimal perioperative antibiotic prophylaxis. Informative posters at point of care and removal of clindamycin and gentamicin from OMRD further reduced inappropriate surgical prophylaxis in patients with PAL.

**Disclosures:**

**All Authors**: No reported disclosures

